# Fine-grained identification of tea plantation parcels in UAV remote sensing images based on DVIT-UNet

**DOI:** 10.1371/journal.pone.0345760

**Published:** 2026-03-31

**Authors:** Yujie Liu, Pengnan Xiao, Yong Zhou, Dali Li, Bochuan Gao

**Affiliations:** 1 Key Laboratory for Geographical Process Analysis & Simulation of Hubei Province, Central China Normal University, Wuhan, China; 2 Institute of Agricultural Resources and Regional Planning, Chinese Academy of Agricultural Sciences, Beijing, China; 3 College of Applied Technology, Hunan Open University, Changsha, China; 4 College of Information Science and Technology, Beijing University of Chemical Technology, Beijing, China; Shandong Agricultural University, CHINA

## Abstract

The complex terrain and diverse management practices in tea-producing regions have resulted in highly fragmented tea plantation plots, posing challenges to precision cultivation, yield estimation, and ecological management. Although remote sensing technology has been increasingly applied to tea plantation mapping, most studies have focused on the overall identification of tea-growing areas, while research on the fine-grained classification and extraction of tea plantation plots within the same spatiotemporal range remains limited. To address this gap, this study proposed a DVIT-UNet model based on ultra-high-resolution unmanned aerial vehicle (UAV) imagery, which integrates a Vision Transformer (ViT) and dilated convolution modules within a UNet framework to effectively capture global semantic dependencies and multi-scale local contextual information. This design specifically targets blurred parcel boundaries, high intra-class heterogeneity, and spectral similarity between tea plantations and surrounding vegetation. Comparative experiments against seven stable deep learning models demonstrated that DVIT-UNet achieved the best performance, with a mean Intersection over Union (mIoU) of 90.48%, F1 score of 94.99%, mean recall (UA) of 94.39%, mean precision (PA) of 95.60%, and a Matthews correlation coefficient (MCC) of 91.13%. Despite its moderate parameter size, the model achieved accurate delineation of small and fragmented tea plots and robustly suppressed false positives in complex backgrounds. The results comprehensively verify the strong capability of DVIT-UNet for fine-grained classification and precise extraction of tea plantation plots from high-resolution UAV imagery, providing a reliable technical foundation for precision tea-plantation management and ecological monitoring.

## 1. Introduction

Tea, cocoa, and coffee are collectively regarded as the three principal global beverage crops [1]. Among these, tea holds particular significance in China, where it has a documented cultural and agronomic history spanning approximately three millennia, with the earliest textual references dating back to the Zhou Dynasty [2]. China’s tea beverage market is now a major sector of the country’s beverage industry, accounting for about 16% of its total market share [3]. Tea is a vital agricultural product and a key source of income in many developing regions. In South China, extensive tea plantations play a vital role in global tea production, and they account for about 40% of the total supply of the world [4]. Over the past few years, however, growing market demand and policy-driven incentives have encouraged the rapid expansion of these plantations. Much of this growth has occurred on steep, high-altitude slopes, and it replaces native forests and farmland that have little or no fertility [5]. Forest loss, habitat fragmentation, reduced landscape connectivity, and deterioration of ecosystem services are ecological factors that change how lands are used [6,7]. While tea cultivation contributes significantly to local livelihoods and regional economies, unchecked expansion threatens environmental stability. Achieving a sustainable balance between economic benefit and ecological protection, therefore, depends on clear regulations that are guided by reliable, high-resolution spatial information. Consequently, there is an urgent need for practical and precise mapping techniques to identify tea plantations at detailed spatial scales, which enable more effective management and environmental evaluation.

Researchers have explored three different methods to map tea-growing areas using satellite images. Traditional dominant approaches rely on classical machine learning, in which spectral metrics, texture measures, and vegetation indices are extracted and then imported into statistical classifiers such as Support Vector Machine (SVM), Random Forests, and decision trees [8–10]. However, these classifiers exhibit limited robustness because classification is adversely affected by spectral confounders, especially when different types of land cover display similar spectra or the same cover exhibits variable spectra. This results in persistent confusion between tea and other woody vegetation and poor performance in heterogeneous mosaics [11,12]. Another line of research exploits hyperspectral or dense multispectral sensors. Informative bands are typically selected by automatic subspace segmentation or dimensionality reduction, while spectral separability is further enhanced by integrating texture descriptors. For instance, hyperspectral studies using Gaofen-5 data have demonstrated that band selection combined with texture features can capture fine spectral differences between tea and arboreal species [13,14]. Nevertheless, these hyperspectral approaches demand complex preprocessing, are vulnerable to the Hughes phenomenon, and depend on specialist dimensionality-reduction expertise; moreover, the availability of high-resolution hyperspectral data is limited, and free sources often lack sufficient spatial detail [15]. Finally, the third stream focuses on deep convolutional architectures that learn hierarchical spectral spatial representations from multi-temporal or multispectral imagery in an end-to-end fashion [16–18]. In particular, when complemented by attention modules and multi-scale fusion, these networks have been shown to generalize well in complex terrain. Yet, despite these advances, boundary fidelity and the detection of small tea parcels remain constrained by the native resolution and radiometric fidelity of the input imagery [19,20]. Therefore, these limitations inspire the exploration of higher-resolution platforms and hybrid methods for parcel-level tea plantation mapping.

To address these limitations, researchers have begun to combine high-resolution multispectral data acquired by low-altitude unmanned aerial vehicles (UAVs) with deep learning techniques. Low-altitude UAV remote sensing, characterized by high maneuverability, strong temporal responsiveness, and fine spatial resolution, has attracted broad interest in the fields of agriculture, forestry, and hydrology [21–23]. For example, in precision agriculture, multispectral sensors mounted on platforms such as the DJI Phantom 4 Pro have been used to produce farm-unit maps with parcel-level accuracy; single-day surveys of complex terrain covering up to 6 km² have been reported, thereby enabling the detection of subtle spectral differences between crops and weeds [24,25]. Compared with conventional satellite imagery, UAV-derived data mitigate spatiotemporal resolution constraints and offer near-real-time, field-scale information [26]. However, traditional UAV workflows that rely on hand-crafted features (e.g., template matching, image binarization) are constrained by operational complexity and poor generalization [27,28]. By contrast, deep learning automatically extracts multi-scale representations and can substantially improve practical performance; for instance, a CGS-YOLO variant based on YOLOv8 achieved a mean average precision (mAP) of 97.2% for maize seedling detection, an improvement of 3.8 percentage points over conventional approaches [29,30]. Convolutional neural networks (CNNs), through hierarchical feature extraction (e.g., edge detection and texture analysis), handle nonlinear problems effectively and excel in tasks such as crop density estimation and object extraction [31,32]. When fused with hyperspectral inputs and attention mechanisms, CNNs can resolve spectral differences between tea and woody species and enhance classification robustness.

In recent years, the Vision Transformer (ViT) has been incorporated into semantic segmentation frameworks [33–35]. Unlike conventional convolutional approaches, ViT employs self-attention mechanisms that explicitly model global dependencies among image points [36,37]. This global receptive field, therefore, facilitates contextual integration across entire plantations, improving the model’s ability to distinguish tea fields from visually similar surroundings, such as shrubs or grasslands [38]. By enhancing global semantic coherence, ViT clearly reduces the inherent limitation of the restricted field of view commonly found in CNN-based encoders [39], thus achieving sound performance in complex remote sensing and agricultural mapping tasks [40]. In the context of tea plantation segmentation, however, capturing fine-grained boundaries and subtle structural details remains equally crucial [41,42]. Conventional CNN downsampling tends to cause the loss of spatial detail, which in turn obscures the accurate mapping of narrow plantation rows and small tea bushes [37]. Dilated convolution, by contrast, provides an elegant remedy; it enlarges the receptive field while preserving feature resolution and introduces no additional parameters [36,43]. By applying multiple dilation rates (d), the model can simultaneously extract multi-scale features, thus detecting both extensive plantation patches and minute structural details [44]. This capability is particularly advantageous for UAV imagery segmentation, where tea plantations typically display intricate row patterns and diverse canopy structures [41].

To overcome the shortcomings of existing tea plantation extraction models [45,46], an ultra-high-resolution extraction approach was developed, in which attention mechanisms and dilated convolutions were embedded into a UNet backbone. The UNet architecture is defined by its symmetric encoder-decoder structure and modular design. Because of its clear connection interfaces, it offers convenient points for integration and is therefore highly extensible. Through the incorporation of the two modules, feature maps at different scales and resolutions were fused and adaptively weighed, allowing the network to better capture complex spatial patterns and variations in tea plantation areas. As a result, information loss during feature extraction was reduced, boundary delineation in high-resolution images became more precise, and sensitivity to small or fragmented tea plots was improved. In this way, accurate and robust semantic tea plantation segmentation of tea plantations was achieved.

## 2. Materials

### 2.1. Study area

**Fig 1:**
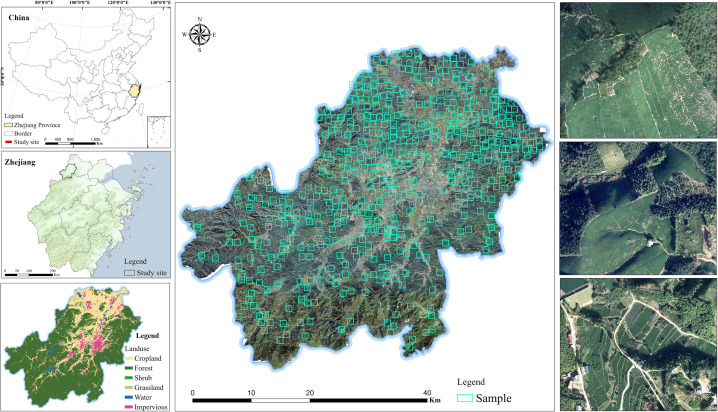
Study area (Anji County).

This study focuses on Anji County of Huzhou City in northwestern Zhejiang Province, China. Lying at the Yangtze River Delta’s center, Anji features a basin-shaped landscape, with elevations sloping down from southwest to northeast(Fig 1). The region is dominated by a subtropical monsoon climate, which provides temperature and precipitation conditions favorable for tea production. The tea industry in Anji constitutes a local economic mainstay. By 2025, tea plantations covered approximately 16,200 ha, accounting for about 8.6% of the county’s land area. Among these, white-tea cultivation occupied roughly 11,333 ha, or about 69.9% of the county’s total tea area. The Anji white tea brand was valued at CNY 5.486 billion in 2024 and has been ranked among China’s leading regional tea brands for fifteen consecutive years. In addition, Anji white-tea processing techniques, recognized as famous intangible cultural heritage assets, have been inscribed on the Representative List of the Intangible Cultural Heritage of Humanity. Moreover, the brand supports tourism and education initiatives (e.g., the Longwangshan Tea Culture Experience Base) and generates substantial cultural and economic benefits for the county.

### 2.2. UAV imagery acquisition and preprocessing

UAV RGB imagery used in this study was obtained during a field campaign conducted from August to November 2023. Data acquisition was conducted using a DJI Matrice 350 RTK rotary-wing UAV equipped with a 1-inch, 20-MP CMOS RGB sensor (90° FOV, aperture f/2.8-f/11). Flights were planned at a nominal altitude of 500 m, yielding imagery with a spatial resolution of 0.2 m per pixel. The platform geolocation accuracy was monitored and determined to be approximately 0.1 m vertically and 0.1m horizontally. All sorties were conducted under stable meteorological conditions (wind speeds of 2–3 m·s ⁻ ¹ and no precipitation); each sortie lasted about 60 minutes and generated six image sets covering the study area. Raw frames were geometrically corrected, orthorectified, and mosaicked; the processed products consisted of three-band (R, G, B) orthophotos referenced to the China 2000 coordinate system (Zone 40 projection).

### 2.3. Image preprocessing

To ensure data quality and reproducibility, the UAV-obtained images were preprocessed following a standard workflow. All raw frames were geometrically corrected, orthorectified, mosaicked, and spatially cropped to the study area. A terrain-residual regression procedure was applied to assess geometric accuracy. RMSE_x and RMSE_y were both < 0.20 m, while the maximum residual was 1.15 m, below the 2.0 m threshold. A Moran’s I assessment of the residuals yielded a p-value of 0.12 (> 0.05), indicating the absence of statistically significant spatial autocorrelation and systematic positional bias. Seam reduction was achieved through a weighted-average compositing algorithm, ensuring smooth transitions between adjacent images. Finally, the county boundary was extracted from the mosaicked product via the Extract by Mask tool in ArcGIS Pro 3.4. The clipped dataset then served as the base images for the tea plot annotations and subsequent deep learning tests.

### 2.4. Manual annotation of UAV imagery

**Fig 2:**
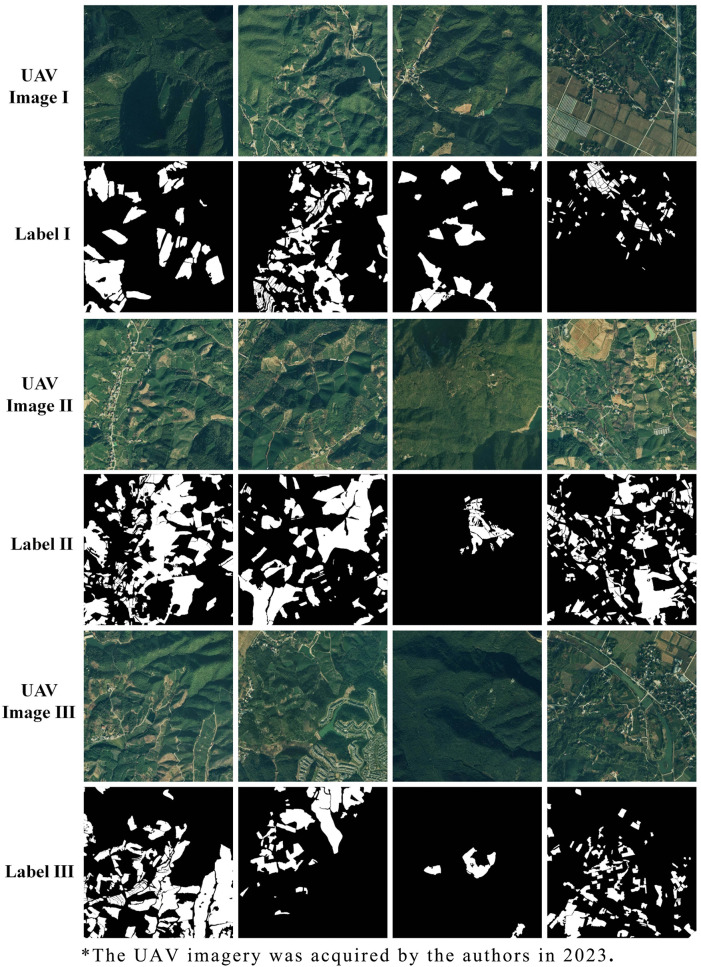
Representative annotations of tea plantations on UAV imagery.

To deliver high positional and thematic accuracies and thus clear plantation boundaries, expert visual interpretation was employed for UAV imagery annotation via the LabelMe software (Fig 2). LabelMe, an interactive image annotation tool proposed by MIT researchers, is dedicated to creating high-quality datasets for computer vision tasks. Its principal advantages include multimodal annotation support (including instance and semantic labeling), flexible data management, and open-source extensibility [47]. The annotations were then compiled into a labeled dataset (tea plantation or otherwise). The labels were stored in JSON files that maintained linkage to the original image, ensuring data traceability. They could also be easily turned into PNG label masks for visualization. Besides, the JSON-formatted annotations facilitated compatibility with various model formats, enabling the use of the same annotation set across workflows.

### 2.5. Training and validation datasets

Dataset preparation was conducted across the study area of Anji County. The UAV images were partitioned into squares with a dimension of 1024 m × 1024 m, initially yielding 642 tiles. After excluding invalid data, we obtained a final dataset of 500 entries. Each tile was then subdivided into 100 512 pixel × 512 pixel patches, yielding ~50,000 basic units. Subsequently, the dataset was divided into training and validation subsets in an 8:2 split. To prevent boundary-induced checkerboard artifacts during mosaicking, no overlap (0%) was applied between adjacent tiles. Finally, standard data-augmentation techniques were applied to the training subset to enhance sample diversity and improve model robustness.

## 3. Methods

### 3.1. Model experiments and evaluation

**Fig 3:**
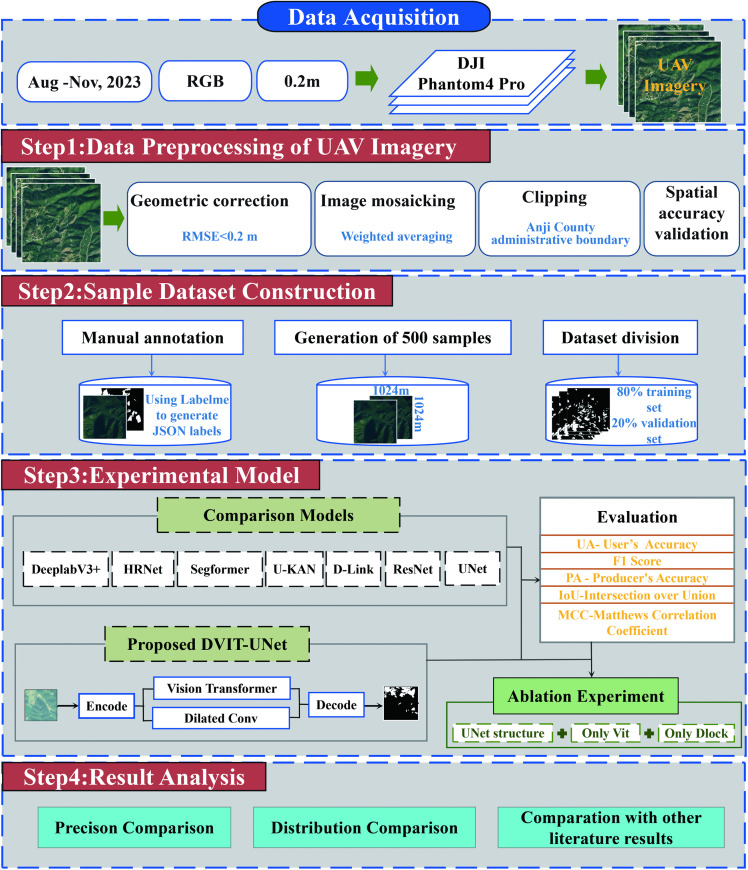
Technical workflow.

Several comparator models were tested, and DVIT-UNet was introduced as the primary model in this study. In addition, ablation studies were conducted to evaluate the individual components’ contributions to overall model performance, quantified using standard evaluation metrics and further benchmarked against previous results. Finally, the accuracy and spatial distribution of predictions were examined and visualized (Fig 3).

### 3.2. DVIT-UNet architecture

**Fig 4:**
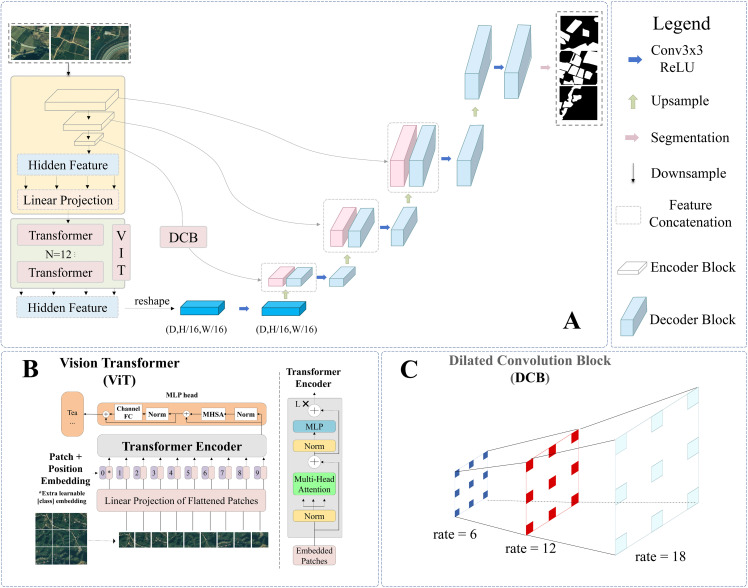
DVIT-UNet network structure.

A deep learning architecture referred to as the Dilated Vision Transformer UNet (DVIT-UNet) was developed to extract tea plantations from UAV imagery. The model follows a U-shaped encoder-decoder paradigm, as illustrated in Fig 4. Its central principle is to integrate dilated convolution with ViT for enhanced feature representation and better overall performance. In the UNet encoder, an attention module and a dilated convolution module are incorporated. The former explicitly establishes long-range dependencies between arbitrary image positions, overcoming CNNs’ restricted receptive field that restrains the capture of global context. Following local feature extraction, the dilated convolution module strengthens the identification of fine-grained details.All code used to implement DVIT-UNet and reproduce the results reported in this study is openly available at GitHub: https://github.com/Ayman1960/DVIT/tree/main.

#### 3.2.1. Encoder and decoder.

The overall network architecture is depicted in Fig 4A. Outputs from the encoder are passed to subsequent stages, where high-dimensional feature representations undergo further processing. The decoder then reconstructs the image by upsampling fused outputs from heterogeneous modules. The model consists of a contraction path (left) and an expansion path (right). A pretrained ResNet-34 served as the feature extractor for the contraction path, with the feature maps produced by each residual block adopted as encoding features. Convolution and pooling operations in ResNet-34 progressively reduce spatial resolution while increasing the channel depth, analogous to the convolution and downsampling steps of the original UNet.

Each step of the expansion path involves upsampling the feature map, followed by a 2 × 2 transposed convolution (i.e., an “up-convolution”) that reduces the number of feature channels by half. The up-convolved output is concatenated with the corresponding cropped feature map from the contraction path, and subsequently processed by two successive 3 × 3 convolutions with ReLU activations. High-resolution detail is reintroduced through skip connections, transferring feature maps from each ResNet-34 stage. Since standard convolution operations reduce spatial extent at the borders, feature maps are cropped to ensure dimensional alignment before concatenation. At the final layer, a 1 × 1 convolution is applied to map each feature channel to the required number of semantic classes. The resulting segmentation map can then be seamlessly tiled (Fig 4A).

#### 3.2.2. Vision transformer block.

The ViT module is illustrated in Fig 4B. In the standard Transformer formulation, the input consists of a one-dimensional sequence of token embeddings. To accommodate two-dimensional imagery, the input image with resolution (H, W) and C channels is divided into non-overlapping square patches of size (P × P). The total number of resulting patches, N, which serves as the effective input sequence length to the Transformer, is thus given by N = HW / P². Each flattened patch is then mapped to a fixed-dimensional vector D through a trainable linear projection, which generates the patch embeddings for the Transformer (Equation (1)). Analogous to the class token in BERT, a learnable classification token is prepended to the patch-embedding sequence. The final state of this token at the Transformer output is used as global image representation y (Equation (4)). During both pretraining and fine-tuning, a classification head is attached to this representation: specifically, a multilayer perceptron (MLP) with one hidden layer is used during pretraining, whereas a single linear layer is adopted during fine-tuning.

Positional information is incorporated into the token sequence through learnable one-dimensional position embeddings, which are added to the token vectors before being fed into the Transformer encoder. The encoder consists of alternating multi-head self-attention layers and MLP blocks. Layer normalization is applied before each block, while residual connections are applied after each block. Each MLP block comprises two linear layers separated by a GELU nonlinearity (Equations (2),(3)). The ViT processing pipeline can be concisely summarized below.


$${𝐳}_{\text{0}}=\left[{𝐱}_{\text{class}};\overset{\text{1}}{\underset{p}{𝐱}}𝐄;\overset{\text{2}}{\underset{p}{𝐱}}𝐄;\ldots ;\overset{N}{\underset{p}{𝐱}}𝐄\right]+{𝐄}_{pos},𝐄\in {\text{R}}^{\left(P^{\text{2}}\cdot C\right)\times D},{𝐄}_{pos}\in {\text{R}}^{(N+\text{1})\times D}$$ (1)

$${𝐳}_{l}^{\prime }=\text{MSA}\left(\text{LN}\left({𝐳}_{l-\text{1}}\right)\right)+{𝐳}_{l-\text{1}},l=\text{1}\ldots L$$ (2)

$${𝐳}_{l}=\text{MLP}\left(\text{LN}\left({𝐳}_{l}^{\prime }\right)\right)+{𝐳}_{l}^{\prime },l=\text{1}\ldots L$$ (3)

$$𝐲=\text{LN}\left(\overset{\text{0}}{\underset{L}{𝐳}}\right)$$ (4)

#### 3.2.3. Dilated block.

Dilated convolution, first noted by Yu et al. [48] for its practical use, has since been explored by many researchers. Its main aim is to expand the receptive field without the reduction of spatial resolution through downsampling, which makes it suitable for multi-scale image segmentation. In the present study, dilation rates (d) of 6, 12, and 18 were applied to capture contextual features of tea plantations at different scales. The smaller rate (d = 6) helps preserve local details, such as tea row boundaries, while the intermediate rate (d = 12) balances local and global contexts. The larger rate (d = 18), in contrast, extends the receptive field to cover broader spatial patterns. By using this progressive design, the network can model both fine-grained structures and large-scale dependencies. At the same time, the potential gridding effect that arises with excessive dilation can be alleviated. This setup follows common practices in semantic segmentation, where d = 6, 12, and 18 are often adopted to achieve reliable multi-scale feature representation [49–51]. Recent studies in UAV-based remote sensing and agricultural mapping also confirm that these d values are effective for delineating crop boundaries and heterogeneous plantation patterns [52–55]. The implementation of dilated convolution in the DVIT-UNet architecture is illustrated in Fig 4C.

### 3.3. Comparison models

To evaluate the proposed model’s performance on tea plantation segmentation, we implemented several canonical and stable models for comparison, including UNet, ResNet, DeepLabV3 + , D-LinkNet, HRNet, SegFormer, and U-KAN.

UNet, a canonical image segmentation model, is characterized by a symmetric U-shaped encoder-decoder architecture. The encoder performs downsampling to extract multi-scale semantic features, while the decoder progressively restores spatial resolution via upsampling. Skip connections directly fuse low-level detail features from the encoder with higher-level semantic features in the decoder, mitigating information loss and improving segmentation accuracy for small objects. This architecture has demonstrated robust performance in small-sample training scenarios [56].

ResNet, a canonical deep neural network architecture, introduces residual connections that enable the network to learn residual mappings between inputs and outputs instead of directly approximating complex target functions. This design substantially simplifies the optimization of very deep networks and mitigates the vanishing gradient problem by facilitating cross-layer gradient propagation. When used as a backbone, the ResNet encoder progressively downsamples feature maps via strided convolutions, reducing their spatial resolution. To restore the resolution required for image segmentation tasks, a decoder module is appended after the ResNet encoder [57]. In this study, transposed convolutions were employed for multi-scale feature fusion and upsampling to ensure that the segmentation outputs match the input resolution.

D-LinkNet, proposed by researchers at Beijing University of Posts and Telecommunications, is a remote sensing image segmentation network designed for complex topological structures and scale variations. Its core is the integration of multi-scale contextual information, achieved using cascaded dilated convolutions that fuse shallow detail features with deep semantic representations. Convolutions with different values of d are used to effectively capture information at multiple scales [58].

DeepLabV3 + has been developed to address some of the challenges in multi-scale feature extraction, and it appears to perform well in this regard. Batch normalization layers were added to the atrous spatial pyramid pooling (ASPP) module, which can help improve optimization stability and support recognition that is less sensitive to scale variations. A global average-pooling operation is also included, and this seems to strengthen the network’s ability to capture broader contextual information. By combining these refinements with an encoder-decoder framework, the allows low-level spatial features to be used with high-level semantic representations. As a result, segmentation boundaries tend to be sharper and more precise, although performance can still vary depending on the complexity of the scene [59].

HRNet keeps high-resolution feature maps throughout feature extraction, and at the same time, it gradually adds parallel low-resolution branches to complement the main high-resolution branch. This approach not only helps the network capture contextual information more effectively but also preserves fine image details, which is particularly important for single-image depth estimation tasks [60].

U-KAN creatively combines the Kolmogorov-Arnold Network (KAN) with the UNet framework, and in doing so, it replaces the fixed activation functions typically used in convolutional and MLP modules with learnable univariate spline functions, which allows the network to dynamically map nonlinear features and better capture complex patterns. At the same time, U-KAN adds tokenized KAN (Tok-KAN) blocks together with depthwise separable convolutions (DSC). This combination helps cut computational cost by roughly half, while also improving segmentation accuracy. Saliency map analysis suggests that U-KAN focuses mainly on target boundaries rather than internal regions, helping to clarify how the network makes its decisions [61].

SegFormer is a simple and efficient Transformer-based semantic segmentation framework that was introduced by NVIDIA. It uses the Mix Transformer (MiT) without positional encoding, which allows it to generate multi-scale features through overlapping patch merging. By doing so, the network preserves high-resolution details while also capturing low-resolution global semantic information, avoiding the performance drops often seen in conventional ViTs due to positional-encoding interpolation. A lightweight decoder then combines multi-level features using only MLPs, removing the need for more complex modules such as dilated convolutions or ASPP. By combining local and global attention, SegFormer achieves efficient segmentation with approximately 50% fewer parameters than comparable architectures, demonstrating strong performance in autonomous driving and satellite image processing applications [38].

#### 3.3.1. DVIT-UNet model training parameters.

The DVIT-UNet model was trained on Windows 10 using Python 3.9 within a PyTorch-based deep learning environment. The initial batch size was set to 30, and the maximum number of training epochs was fixed at 100. Model optimization was guided by the metrics of *dice loss_average* and *mcc_average*, and training was halted automatically when optimal performance on the monitored validation metrics was achieved.

To enhance training stability and convergence, a **Reduce**LRO**nPlateau** learning rate scheduling strategy was employed to dynamically adjust the optimizer’s learning rate based on validation loss behavior. The initial learning rate was set to **2** × **10** ⁻ **⁴**. When the validation loss failed to improve for **two consecutive epochs (patience** = **2)**, the learning rate was reduced by a factor of **0.5**. A minimum learning rate threshold of **5** × **10** ⁻ **⁶** was enforced to prevent excessively small updates. The scheduler was configured in **verbose mode**, enabling logging of learning rate adjustments during training.

To ensure experimental reproducibility, a **fixed random seed of 41** was used for all experiments, including data shuffling, weight initialization, and training procedures. Model weights were initialized using the **default PyTorch initialization schemes**. Specifically, weights of convolutional and fully connected layers (*nn.Conv2d*, *nn.Linear*) were initialized using **Kaiming Uniform (He Uniform) initialization**, with biases set to zero. For *nn.BatchNorm2d* layers, scale parameters (γ) were initialized to 1 and shift parameters (β) to 0. For recurrent layers such as *nn.*LSTM and *nn.*GRU, weights were initialized using a uniform distribution, and biases were set to zero.

#### 3.3.2. Training parameter settings for comparative models.

For performance benchmarking, seven representative semantic segmentation networks—UNet, ResNet, DeepLabV3 + , D-LinkNet, HRNet, SegFormer, and U-KAN—were implemented for comparative analysis. Most comparison networks were CNN-based; however, SegFormer is transformer-based, and U-KAN integrates nonstandard tokenized KAN modules within a U-Net-like convolutional backbone, so the set includes both convolutional and non-convolutional architectures.

To ensure experimental fairness, identical training, validation, and testing datasets were used for all models, including DVIT-UNet. Model training was conducted on an NVIDIA Quadro P4000 GPU using the PyTorch framework. Convolutional layers were initialized with weights drawn from a Gaussian distribution (mean = 0, standard deviation = 1 × 10 ⁻ ⁶). We trained the models using stochastic gradient descent (SGD), with an initial learning rate of 0.0005. A learning rate scheduling mechanism was applied during training to maintain convergence stability. Each model was trained for a maximum of 100 epochs with a batch size of 30.

### 3.4. Ablation study design

To evaluate the contribution of each component to tea plantation segmentation, an ablation experiment was designed. Three model variants were considered: one employing only the Vision Transformer (OnlyViT), another using only the dilated convolution block (OnlyDlock), and a third combining both. Therefore, the setup allowed direct observation of each component’s effect under comparable conditions. Therefore, each variant was trained, validated, and tested on the same dataset as the complete DVIT-UNet model under identical hyperparameter configurations, preprocessing steps, and hardware settings.

### 3.5. Evaluation metrics

Model performance was evaluated using well-established semantic segmentation metrics, including precision (PA, or producer’s accuracy), recall (UA, or user’s accuracy), the F1 score, intersection over union (IoU), and the Matthews correlation coefficient (MCC). PA (Equation 6) is, however, calculated as the proportion of true positives (TPs), thereby reflecting how reliably the model identifies target regions. Recall (UA), by contrast, compares TPs to all actual positives (Equation 7) and indicates how completely the model captures the relevant instances. The F1 score (Equation 8), which is the harmonic mean of PA and UA, balances the two metrics and becomes particularly useful when class distributions are uneven. IoU (Equation 9) shows how much the predicted regions overlap with the reference regions. A value of 0 means there is no overlap, while 1 indicates a complete match. MCC, on the other hand, combines all four entries of the confusion matrix into a single value. This provides a reliable measure of performance, even when the data are highly imbalanced; its values range from −1, representing total disagreement, to +1, indicating perfect concordance.indicating perfect concordance.


$$MCC=\frac{TP\times TN-FN\times FP}{\sqrt{(TP+FP)\times (TP+FN)\times (TN+FP)\times (TN+FN)}}$$ (5)

$$\;Precision(PA)=\frac{TP}{TP+FP}$$ (6)

$$Recall(UA)=\frac{TP}{TP+FN}$$ (7)

$$F\text{1}=\frac{\text{2}*Precision*Recall}{Precision+Recall}$$ (8)

$$IoU=\frac{TP}{TP+FP+FN}$$ (9)

Here, TP is the number of instances in which both the predicted and reference labels are positive, FP represents the quantity of cases that are predicted to be positive despite a negative reference value, TN is the number of instances where both the predicted and reference labels are negative, and FN counts instances predicted as negative despite a positive reference value.

## 4. Results

### 4.1. Comparative accuracy of different models in tea plantation segmentation

#### 4.1.1. Training efficiency.

**Fig 5:**
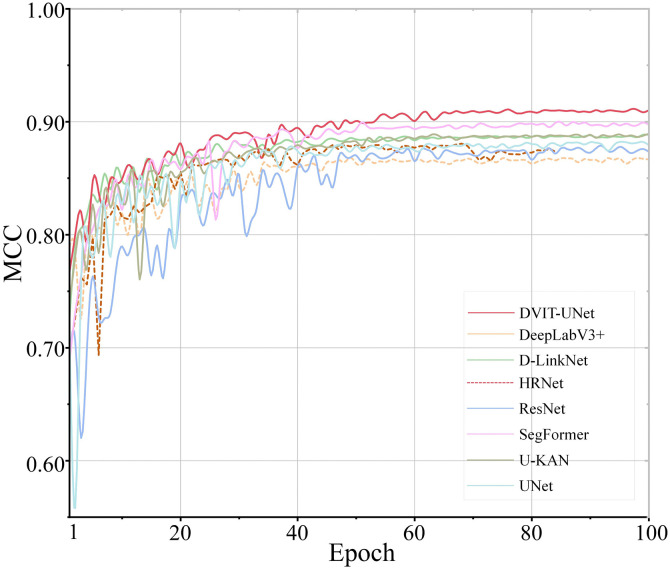
Comparative analysis of MCC accuracy for various models.

As shown in Fig 5, all models underwent a rapid escalation in MCC during the early training period (the first 10 epochs); nonetheless, this surge was accompanied by considerable volatility. Notably, ResNet experienced a sharp drop to 0.30 at epoch 2; however, it recovered on time, thus revealing its initial instability. These fluctuations are mostly due to an overly aggressive learning rate, which can, in turn, provoke numerical oscillations. In other words, the model’s early performance instability is likely from this parameter choice. Hence, previous studies recommend employing a learning rate warm-up, which, by so doing, can reduce these oscillatory behaviors and stabilize training dynamics.

In contrast, DVIT-UNet and SegFormer began with comparatively high MCC scores and showed a consistent upward trajectory during the initial stages, exhibiting minimal fluctuations. Their stability can be attributed to strong architectural designs and the use of pretrained backbones.

Between epochs 10 and 50, the MCC curves for all models began to plateau, which indicates that they were nearing convergence. DVIT-UNet and SegFormer keep their leading positions, and they show only minor improvements on the MCC value, which is high already. In contrast, D-LinkNet and U-KAN continued to make steady and gradual progress, while DeepLabV3+ and HRNet achieved only small gains. The slightly higher MCC observed for HRNet can likely be attributed to its parallel multi-scale convolutional design, which, in turn, helps to preserve semantic features across different spatial resolutions. ResNet and U-Net, however, progress at a slower rate, lagging behind the other models.

In the late training phase of epochs 50 and 100, most models had essentially stabilized. DVIT-UNet achieved the highest MCC of 0.92, with a smooth and steady curve that reflected both rapid convergence and reliable performance. SegFormer followed closely and reached roughly 0.90, while D-LinkNet and U-KAN also maintained strong results. DeepLabV3 + finished with the lowest MCC, ResNet displayed uneven convergence, and U-Net finished with smaller final scores, followed by a higher variability or fluctuations.

DVIT-UNet performs better mainly because of two architectural improvements. The first improvement is the use of dilated convolutions, which widen the receptive field and help the model to gather richer contextual details across multiple scales and not losing spatial accuracy. The second improvement is its attention mechanism, which allows the network to focus on the most relevant features while reducing background interference. Together, these elements enhance segmentation accuracy and make the training process more stable. By so doing, DVIT-UNet becomes particularly effective for complex and high-resolution remote sensing imagery.

#### 4.1.2. Recognition accuracy of different models.

The statistical significance tests(Table 1)demonstrated that the DVIT-UNet outperformed all comparison models, including DeepLabV3 + , D-LinkNet, HRNet, ResNet, SegFormer, UNet, and U-KAN. Specifically, the pairwise tests yielded p-values ranging from 0.0193 to 0.0488, all of which were below the conventional 0.05 significance threshold, thereby confirming that the performance improvements of DVIT-UNet were statistically significant at the 95% confidence level. These results provide robust evidence that the integration of dilated convolution and ViT mechanisms in DVIT-UNet offers clear advantages over both classical CNN-based architectures and other recent transformer-based segmentation networks.

**Table 1:** Significance test results of DVIT-UNet accuracy.

Model	DeepLabV3+	D-LinkNet	HRNet	ResNet	SegFormer	UNet	U-KAN
**P**	0.0488**	0.0221**	0.0211**	0.0193**	0.0206**	0.0199**	0.0206**

Experimental comparisons (Table 2) reveal that DVIT-UNet attained the highest performance across all principal evaluation metrics, all of which exceeded 0.90. Its F1 score of 0.94 exceeded those of SegFormer and D-LinkNet by approximately 0.62% and 0.92% respectively. For MCC, DVIT-UNet achieved 0.91, outperforming SegFormer (0.8998) and D-LinkNet (0.8950) by margins of approximately 1.14% and 1.62%, respectively. Similarly, the IoU score of 0.90 exceeded SegFormer (0.89) and D-LinkNet (0.88) by approximately 1.24% and 1.82%, respectively. These differences confirm DVIT-UNet’s leading position, with advantages of 1% plus in the F1 score, MCC, and IoU metrics over the best-performing baseline.

**Table 2:** Comparison of the final accuracy across models.

Model	Accuracy (%)					Comparison accuracy (%)				
	UA	PA	F1 Score	MCC	IoU	UA	PA	F1 Score	MCC	IoU
DVIT-UNet	94.39	95.6	94.98	91.12	90.47	–	–	–	–	–
DeepLabV3+	88.29	97.35	92.57	86.8	86.22	8.68	−3.12	3.26	5.7	5.71
D-LinkNet	92.92	95.29	94.06	89.5	88.85	1.47	0.31	0.92	1.62	1.62
HRNet	92.32	95.02	93.62	88.72	88.07	2.07	0.58	1.36	2.4	2.4
ResNet	91.87	94.47	93.11	87.87	87.19	2.52	1.13	1.87	3.25	3.28
SegFormer	93.47	95.29	94.36	89.98	89.36	0.92	0.31	0.62	1.14	1.11
UNet	92.58	94.91	93.7	88.87	88.22	1.81	0.69	1.28	2.25	2.25
U-KAN	92.57	95.01	93.74	88.93	88.28	1.82	0.59	1.24	2.19	2.19

SegFormer yielded results most similar to those of DVIT-UNet (within 1.2% across all metrics); however, it remained less effective at delineating fine boundaries (e.g., tea plant edges). D-LinkNet and U-KAN (IoU = 0.88) achieved comparable performance, suggesting diminishing returns from current architectural adjustments for fine-grained target extraction. Traditional CNN-based models, such as ResNet (IoU = 0.87), generally lack the mechanisms that would balance global semantic representation and fine detail preservation. Although the highest PA value was achieved by DeepLabV3 + , which is 0.98, its UA value of 0.85, however, indicates that the system missed a large proportion of TPs. In contrast, DVIT-UNet achieved a UA of 0.9439, and it has a stronger recall for the primary target class.

Overall, DVIT-UNet achieved superior final performance and a smooth training trajectory. Baseline models, such as ResNet, which appeared more sensitive to initialization and hyperparameter selections, however, can lead to slower and less stable convergence. These discrepancies, therefore, reveal fundamental contrasts in both model architecture and representational depth. SegFormer, by integrating Transformer-based attention with dilated convolution, efficiently extracted richer, high-quality feature representations. HRNet, through its cross-scale information exchange, was able to preserve high-resolution features across multiple layers and was, however, advantageous for image segmentation. Models that combine pretrained convolutional backbones (for instance, DeepLabV3+ and HRNet) would likely converge more quickly and with stability, while those that lack such enhancements (e.g., ResNet and U-Net), in contrast, displayed inferior performance, slower optimization, and greater fluctuations. Ultimately, architectures like DVIT-UNet nearly achieved an MCC of 1, which demonstrates a very accurate classification for both positive and negative samples. Models such as ResNet, however, produced noticeably lower values of MCC, which underscores their weaker discriminative capacity.

### 4.2. Comparative analysis of model performance

**Fig 6:**
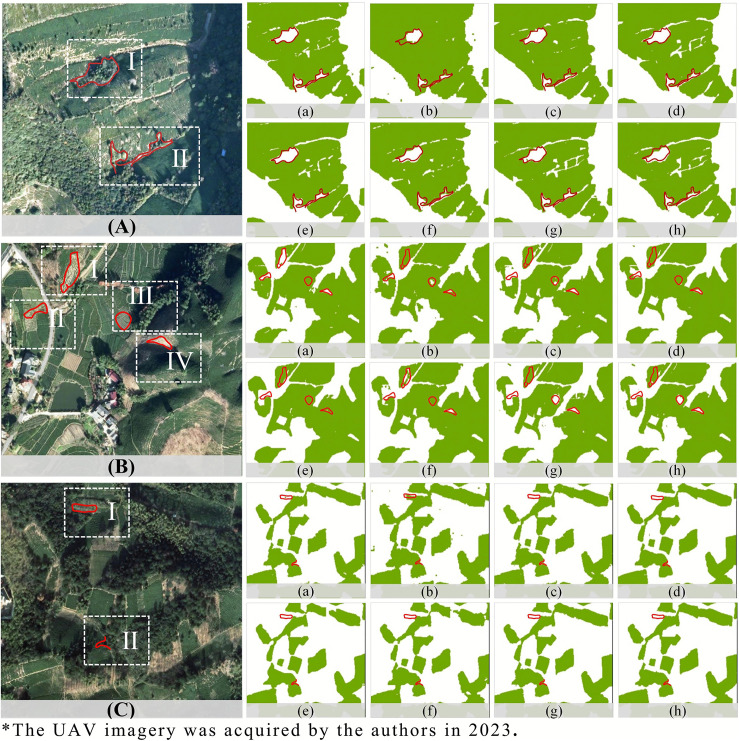
Comparison of prediction maps: (a) DVIT-UNet, (b) DeepLabV3 + , (c) D-LinkNet, (d) HRNet, (e) ResNet, (f) SegFormer, (g) U-Net, and (h) U-KAN.

The visual analysis that shows how DVIT-UNet differentiated tea plantations from arbor forests is shown in Fig 6A. The model accurately detected arbor forest patches within tea-growing landscapes (Fig 6A-I) and precisely delineated the boundaries between them (Fig 6A-II). This substantially higher boundary identification precision underscores DVIT-UNet’s enhanced boundary-preserving segmentation ability.

As shown in Fig 6B, DVIT-UNet demonstrated a consistent and accurate performance in detecting bare soil, sparse tea plantations, and dense tea plantations. Regions such as Fig 6B-I, Fig 6B-II, and Fig 6B-IV, which exhibited very sparse tree coverage, were correctly classified as bare soil. Furthermore, adjacent sparse tea plantations (e.g., Fig 6B-III) were accurately identified by DVIT-UNet, whereas competing models either misclassified them as non-tea areas or achieved only partial detection.

In Fig 6C, DVIT-UNet exhibited minor noise artifacts at location C-I, indicating that although the model detected the presence of the tea plantation, full segmentation was not achieved. Under these conditions, D-LinkNet and DeepLabV3 + also successfully detected the same tea plantation block, whereas several other models failed to do so. This region was heavily affected by shadows cast by surrounding arbor trees, which likely degraded detection accuracy. Notably, DeepLabV3 + achieved high PA in this case but produced coarsely defined boundaries and spurious noise points, whereas D-LinkNet, leveraging its multi-layer dilated convolution structure, captured the tea plantation block with moderate detail.

Overall, models such as D-LinkNet, HRNet, ResNet, UNet, and U-KAN displayed comparable recognition patterns, struggling to distinguish bare soil from sparse tea plantations and sparse tea plantations from arbor forests. SegFormer delivered competitive results but produced coarser boundaries and occasional gaps compared to DVIT-UNet.

### 4.3. Comparative analysis of ablation study outcomes

**Fig 7:**
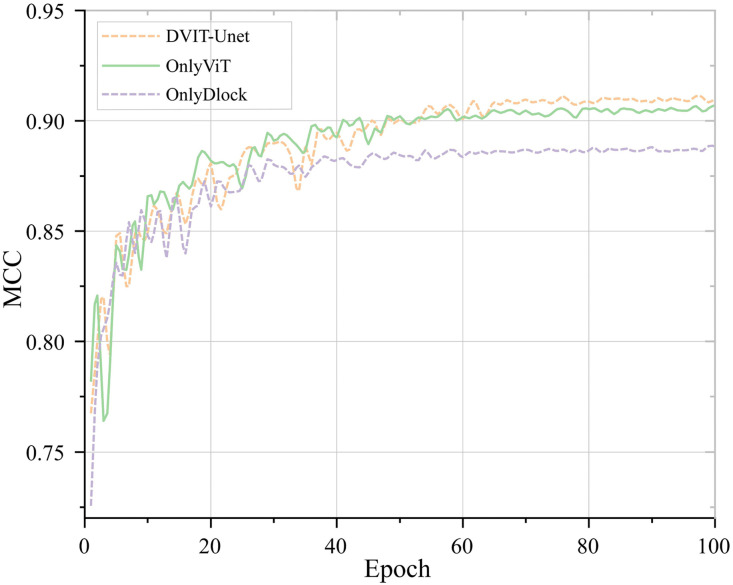
Comparison of MCC levels in the ablation study.

The MCC values increased steadily for all the models throughout training, as shown in Fig 7, which suggests that the networks gradually learn to extract more discriminative features over time. However, despite this overall upward trajectory, the models differed notably in their rates of improvement, degrees of fluctuation, and final MCC outcomes.

The two configurations that combine the attention mechanism made it to improved more rapidly in the initial training stage, whereas OnlyDlock exhibited a contrasting, slower, and more gradual rise due to its exclusive dependence on dilated convolutions. Within the first 20 epochs, the values of MCC for OnlyViT and DVIT-UNet rose from roughly 0.80 to 0.88, while OnlyDlock increased more slowly, approaching 0.84 during the intermediate stage. This pattern aligned, therefore, with prior findings [62], which indicate that attention modules enhance the focus of the model on task-relevant features and, by so doing, improve convergence efficiency.

Between epochs 21 and 50, the MCC continued to increase for DVIT-UNet and OnlyViT, and they both exceeded 0.90, while OnlyDlock seemed to plateau around 0.88. The difference between OnlyViT and OnlyDlock suggests that attention mechanisms play a major role in segmentation accuracy, even though dilated convolution still helps by widening the receptive field and capturing features at multiple scales. When combined in DVIT-UNet, these mechanisms appeared to reinforce each other rather than simply adding up, which may explain why this configuration achieved the highest MCC.

Regarding convergence, OnlyDlock stabilized fairly early and roughly by epoch 50, which showed little fluctuation, but its overall accuracy remained limited. DVIT-UNet and OnlyViT reached 0.90 sooner, around epochs 30 and 40, respectively. Their curves were somewhat more variable; however, the variability seemed to reflect faster learning and better generalization.

Overall, the highest MCC value across the early, intermediate, and final training stages was achieved by DVIT-UNet, which shows that its dual-module architecture successfully combines complementary strengths to enhance feature extraction and multi-scale information. The attention mechanism contributed more substantially to performance gains, whereas the dilated convolution module expanded the contextual receptive field, thereby facilitating more effective attention mapping.

The complete architecture surpassed its single-module counterparts, achieving a UA value of 0.9439, an MCC value of 0.9113, and an IoU value of 0.9048. These results affirm the critical role of multimodal feature integration in complex semantic segmentation. The subsequent sections examine this advantage through two complementary aspects.

#### 4.3.1. Global-local feature complementarity.

**Fig 8:**
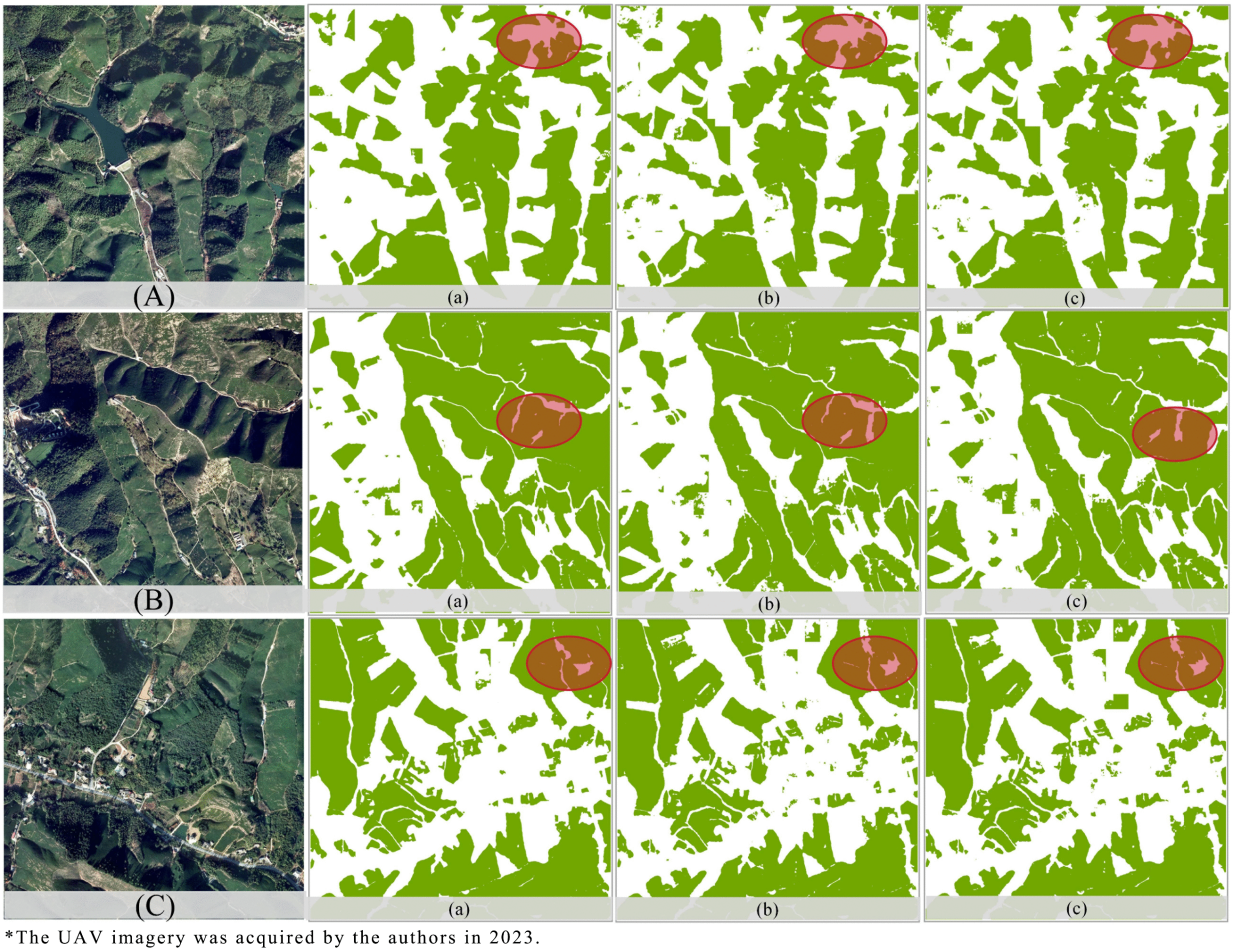
Comparative results of ablation experiment details: (a) DVIT-UNet, (b) OnlyViT, (c) OnlyDlock predicted map.

According to Table 3 and Fig 8, OnlyViT (UA = 0.9378) demonstrated a strong capability to capture global semantic features via multi-head self-attention, thereby effectively modeling large-scale spatial structures, including agricultural land distribution and urban morphologies. Its ability to detect localized fine structures, however, such as narrow transportation corridors or building edges, remained limited. This is attributed to the fixed patch-size partitioning strategy, which, by smoothing high-resolution details during tokenization, reduces sensitivity to fine spatial features.

**Table 3:** Accuracy comparison in ablation study (%).

Model	UA	PA	F1	MCC	IoU
DVIT-Unet	94.39	95.6	94.99	91.13	90.48
OnlyViT	93.78	95.8	94.77	90.71	90.09
OnlyDlock	92.92	95.29	94.06	89.5	88.85

By contrast, the dilated convolution module established hierarchical receptive fields with dilation rates of d = 6, 12, and 18, which enable the robust multi-scale local-context extraction. For example, a smaller dilation of d = 6 will enhance the sensitivity to texture-level details, while a larger dilation of d = 18 will facilitate the recognition of medium-scale objects. When applied independently, this module achieved lower value than OnlyViT. The combined approach, however, further increased the UA value to 0.9439 and raised the IoU value by 0.39%, thus demonstrating that the integration of global and local features produces a more comprehensive and accurate multi-scale object representation for satellite image interpretation.

#### 4.3.2. False-positive suppression and robustness to class imbalance.

Although OnlyViT achieved the highest PA value of 0.9580, it, however, exhibited the lowest UA value of 0.9378, which indicates that a certain degree of over-detection occurred, in which background pixels were, at times, incorrectly classified as part of the target class. This shortcoming was, however, alleviated through the use of dilated convolution, which adaptively adjusted the receptive field according to the dilation rate (d). A larger dilation of d = 18 was applied to the shallow layers to suppress the broad noise patterns, while a smaller dilation of d = 6 was applied at the deeper layers to retain the essential spatial information.

The integration of complementary feature scales facilitated cross-scale feature refinement and allowed the combined model to increase the UA value by 0.61%, while sacrificing only 0.20% of the PA value. This trade-off, however, reflects a more balanced performance, which indicates that the model became better at suppressing false positives (FPs), particularly in images dominated by extensive background regions. The hybrid architecture also achieved an MCC value of 0.9113 and an IoU value of 0.9048, which outperforms each component. The MCC underscores the resilience of the model to imbalanced class distributions and its capacity to maintain discrimination under challenging conditions.

## 5. Discussion

### 5.1. DVIT-UNet performance and internal feature analysis for tea plantation classification

Compared with existing studies, the DVIT-UNet model ranks among the leading methods in terms of accuracy of tea plantation recognition via remote sensing imagery. On 0.2 m UAV imagery, DVIT-UNet achieved an F1 of 0.95, IoU of 0.90, and overall accuracy (OA) of 94%, which approaches or even exceeds the results reported for lower-resolution datasets. Although a temporal deep learning model constructed on Sentinel-2 achieved OA 95.3% [63], the spatial resolution of UAV imagery was substantially higher than that of GF-2 (1 m) and Sentinel-2 (10 m), highlighting the advantage of UAV data for fine-scale mapping. The DVIT-UNet thus demonstrates pronounced advantages in high-resolution imagery applications for the extraction of small tea plots and field boundaries. In addition, an MCC of 0.911 indicates that classification performance, accounting for class balance through the confusion matrix, is similarly high.

Moreover, with respect to classification capability, conventional studies have commonly merged tea plantations with all other land covers into two broad classes, and only a few studies have successfully distinguished arboreal forest from tea plantations. Specifically, as noted by Yin et al., although Sentinel-2 time series can separate vegetation types to some extent, low spatial resolution can lead to “different-objects-same-spectrum” and “same-object-different-spectrum” problems, often causing forests to be misclassified as tea plantations [64]. Furthermore, while Google imagery can exploit texture to distinguish tea plantations from forests, striped features, such as terraced fields, may still be misclassified as tea plantations.

**Fig 9:**
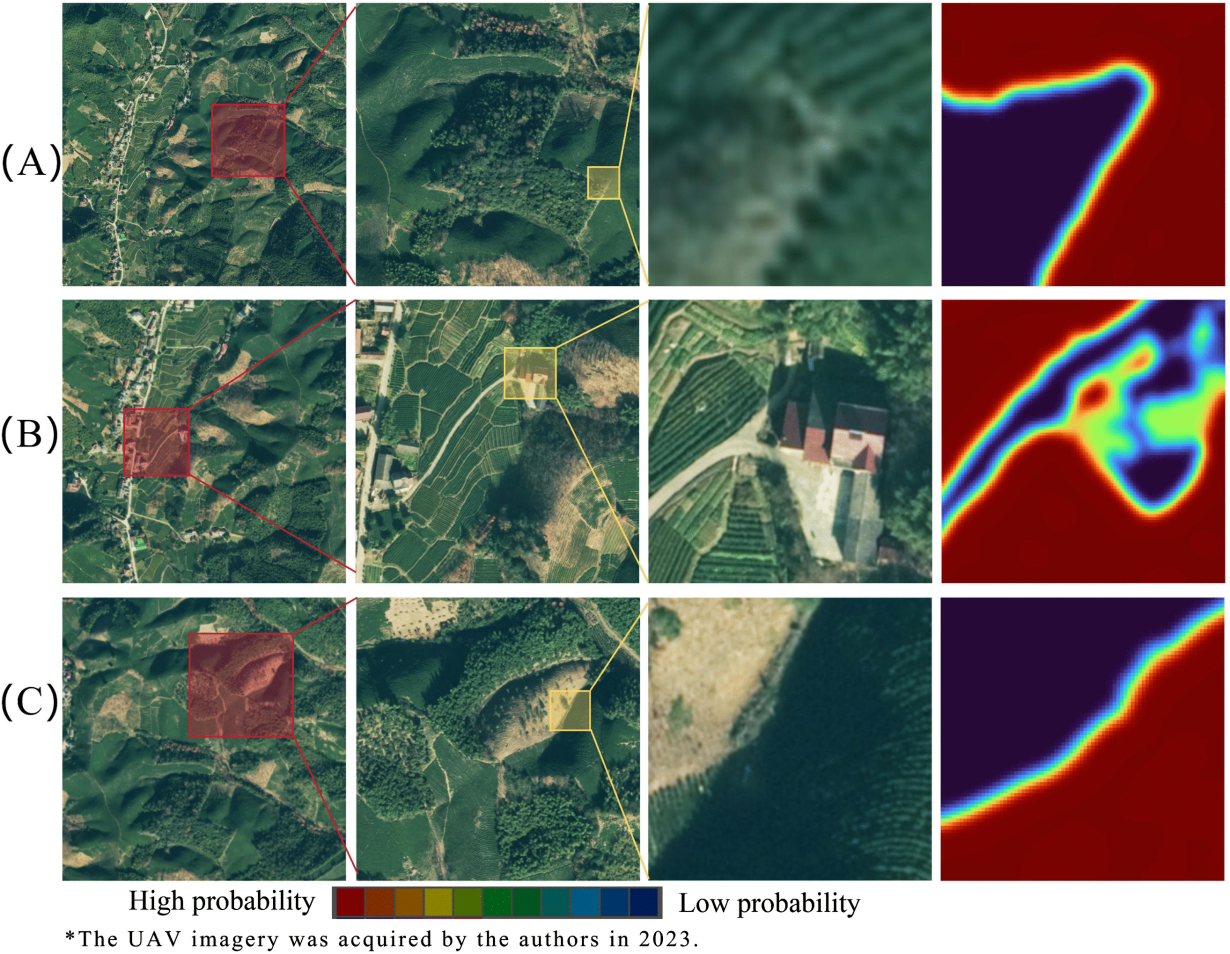
Predicted probability maps for tea plantation recognition.

To demonstrate the model’s internal behavior more clearly, heatmaps were produced based on the gradient magnitudes returned by the final feature extraction layer of the model. This step was applied to three types of images, namely, tea plantations versus bare soil, arborous forest, roads, and other land covers. In these heatmaps, pixels predicted to have a higher probability of being classed as tea plantations are shown in red, whereas those corresponding to other classes are shown in blue (Fig 9). These heatmaps further demonstrate that the DVIT-UNet model employed in this study is successful at delineating tea-non-tea boundaries: transitions from red to blue are abrupt, and only a small number of pixels exhibit gradual change, indicating a clear separation between tea plantations and adjacent non-tea land covers.

The model also demonstrates concentrated identification within tea plantations: the regions identified as tea plantations typically present two similar red tones, both of which are deep and show very little gradation, suggesting that tea rows and inter-row access paths are collectively recognized as a textured whole. These findings indicate that the model relies heavily on the tea plantation texture as a discriminative feature, and the misclassification risk is low within tea areas. This enables more accurate delineation of plantations and reliable discrimination between dense and sparse plantings within the same plantation, which highlights the advantages of the proposed approach for fine-grained multi-class classification and narrows a critical gap in existing literature.

In previous studies, various approaches have been adopted with respect to model architecture and complexity. For example, a traditional machine learning algorithm (random forest) driven primarily by hand-crafted multidimensional features was employed by Zhu et al. [8]. Similarly, deep networks incorporating recurrent units (RNNs) were adopted by Yao, Yin, and Wirabudi et al., which enabled joint spatiotemporal feature extraction by combining 1D-CNN with a UNet-style framework [59]. In addition, an improved UNet variant has been used to process high-resolution orthophotos [65].

In the present study, the DVIT-UNet model—classified as a complex deep learning architecture—was employed to simultaneously enhance long-range dependency modeling and multi-scale feature extraction. Considering network complexity, DVIT-UNet is more elaborate than conventional CNNs or a plain UNet; however, this additional complexity facilitates the capture of fine-scale tea crowns present in very-high-resolution imagery. Although increasing model complexity can lead to improvements in accuracy, these gains, however, come with higher demands for both training data and computational resources. It is therefore important to find a balance between generalization capability and computational efficiency. By doing so, the model remains both practical and reliable. In other words, DVIT-UNet combines multiple architectural techniques, which leverage the high resolution of UAV images. It achieves not only superior accuracy but also fine-grained classification, thus improving overall performance.

### 5.2. Comparison of deep learning architectures for high-resolution tea plantation segmentation

There were, however, notable differences among the competing models when segmenting tea plantations. With respect to boundary delineation, HRNet and DVIT-UNet produced the most accurate descriptions of tea-crown edges due to the preservation of high-resolution feature representations and the use of attention mechanisms. In contrast, models such as DeepLabV3+ and ResNet exhibited comparatively blurred boundaries after upsampling, likely resulting from repeated downsampling operations followed by simple interpolation. However, UNet and UKAN benefited from skip connections and were able to recover some edge information, though their edge segmentation performance was only moderate.

Regarding speckle noise and isolated FPs, DVIT-UNet outperformed the other architectures [66]. The attention modules implemented within DVIT-UNet reinforced spatial and semantic continuity across homogeneous regions, reducing isolated misclassifications [67]. In contrast, classical architectures such as ResNet and DeepLabV3 + produced more scattered noise and spurious detections.

Regarding fine-structure representation, HRNet and DVIT-UNet exhibited superior discriminative capability for small internal tea-plot structures in particular (for example, clusters of tea plants with different densities). HRNet’s multi-scale parallel branches maintained local detail, while DVIT-Unet combined dilated convolutions with attention to effectively capture global context while preserving spatial resolution. SegFormer delivered robust global-context capture and structural consistency, but, in some cases, its representation of extremely fine textures was slightly smoothed [64,68].

While previous hybrid models such as SegFormer and U-KAN have combined CNNs with attention mechanisms, DVIT-UNet distinguishes itself by explicitly enhancing both global context modeling and fine-grained feature extraction through the combination of a ViT-based attention module and a dilated convolution module in the encoder.A comparison of computational efficiency and performance under high-resolution UAV imagery conditions is provided in Table 4. DVIT achieves a throughput of 8.96 million pixels(MPix) per second for 100 patches, which is lower than lightweight models such as ResNet or UNet, reflecting the increased computational cost of the transformer-based module. However, this design yields superior feature representation and accuracy for fine-grained segmentation of tea plantations. Notably, DVIT consumes 487.7 MB of GPU memory and 1744.0 MB of CPU memory, which is higher than most CNN-based models but comparable to other hybrid networks. SegFormer and U-KAN, while more lightweight in terms of GPU usage (103 MB and 156.8 MB, respectively), show lower throughput or accuracy for high-resolution UAV imagery in our experiments.

**Table 4:** Computational Efficiency and Resource Consumption Comparison of Different Models.

Model	Total Time (s)	Average Time per Patch (ms)	Throughput (MPix/s)	Peak GPU Memory (MB)	Memory Usage (MB)
ResNet	0.50	5	23.50	113.8	1171.6
UNet	0.60	6	19.78	256.6	1207.2
DeepLabV3+	0.70	7	18.44	202.3	1409.3
SegFormer	0.70	7	18.43	103.0	1276.3
D-LinkNet	0.70	7	17.41	154.1	1323.1
U-KAN	1.10	11	11.91	156.8	1320.5
DVIT-UNet	1.50	15	8.96	487.7	1744.0
HRNet	2.90	29	4.88	110.9	1251.6

Thus, DVIT-UNet achieves a balance between architectural innovation and performance under challenging high-resolution conditions, offering improved segmentation capabilities at the cost of increased computational requirements. These results underscore the trade-offs inherent in transformer-based hybrid architectures and highlight the advantages of integrating dilated convolution for capturing fine-scale details.

Overall, the MCC convergence curve reached its peak value for DVIT-UNet, indicating that its overall segmentation performance surpassed that of the other models. The principal advantage of DVIT-UNet, however, lies in its combination of attention mechanisms and dilated convolutions. Attention modules, whether channel- or spatial-based, can assign greater weights to salient semantic features, thereby enhancing responses at boundaries as well as within target regions. By so doing, the model achieves a marked improvement in boundary clarity. Dilated convolutions expanded the receptive field without reducing feature map resolution, enabling the network to capture richer multi-scale context under a certain parameter budget. The conjunction of these two components allowed DVIT-UNet to attend to both local details (edges and textures) and global context, resulting in precise, fine-grained segmentation outcomes. The attention mechanism also provided noise suppression that reduced isolated misclassifications, a critical function for mitigating the speckle-like noise common in very-high-resolution tea plantation imagery.

### 5.3. Residual analysis and fine-grained classification performance of DVIT-UNet

**Fig 10:**
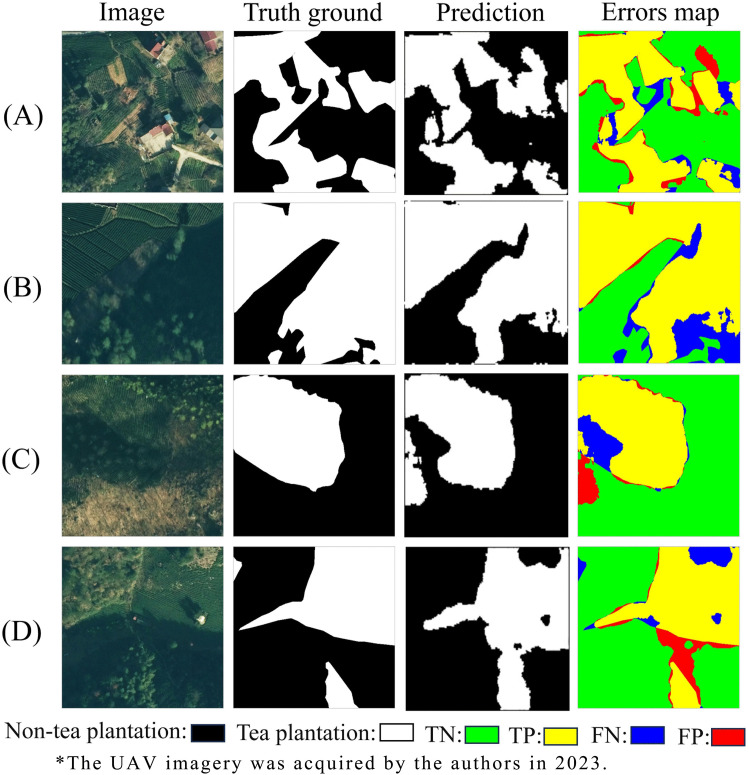
Comparison of residual analysis results.

Residual analysis was employed to visualize the prediction outcomes. The left panel of Fig 10 shows the original remote sensing images and ground truths, while the right panel overlays the model predictions alongside the residuals. Red parts denote regions where prediction errors exceed the threshold, while blue regions represent unpredicted areas. Analysis across multiple samples revealed that prediction errors clustered primarily in two regions: cloud-shadow boundaries and small bare-earth patches. The limited information contained in single-date images was identified as the primary contributor to these elevated misclassification rates.

Several potential strategies could be explored to mitigate these limitations in future work [69–71]. First, the integration of multi-temporal UAV imagery may help reduce shadow-related errors by leveraging complementary illumination conditions across different acquisition times. Second, explicit shadow detection and removal or illumination normalization preprocessing could be incorporated prior to model inference to improve feature consistency. Additionally, multi-scale feature fusion strategies or auxiliary supervision focusing on small-object representation may further enhance the discrimination of fragmented bare patches.

These improvements, however, may introduce additional computational costs. As shown in the efficiency analysis, DVIT-UNet already requires higher inference time and memory consumption compared to lightweight CNN-based models. Incorporating multi-temporal data or additional preprocessing steps would further increase computational complexity and memory usage. Therefore, a balance must be carefully considered between segmentation accuracy in challenging regions and computational efficiency, particularly for large-scale or real-time UAV applications.

A key distinguishing feature of the present model is the combination of very-high-resolution data with a deep network architecture, enabling fine-grained tea plantation classification. First, 0.2 m UAV imagery was employed, offering spatial detail that far exceeds that of Sentinel-2 (10 m) and GF-2 (1 m) imagery, which in turn greatly enhanced object-level details. This improved spatial fidelity enabled clearer boundary distinctions between tea plantations and heterogeneous neighboring covers such as arborous forest and wasteland, mitigating the “different-objects-same-spectrum” and “same-object-different-spectrum” problems typical of coarse-resolution satellite data. In addition, the proposed DVIT-UNet architecture combines dilated convolutions with attention mechanisms, therefore improving the ability of the network to capture multi-scale textures and long-range semantic relationships. According to the present findings, this design effectively differentiated tea plantations from nearby wooded or bare areas, even in cases where textures or edges appear the same. This development goes beyond earlier research that mostly focused on coarse parcel extraction, producing a finer classification granularity that can differentiate tea plots from non-vegetated surfaces and wasteland from sparse tea planting.

Lastly, the model outperformed deep learning techniques and multisource fusion methods applied to Sentinel-2 in terms of identification accuracy at the fine scale. These findings, however, show that the current work provides a promising technical strategy for accurate mapping and management of tea plants by achieving improved classification granularity and accuracy for high-resolution photos.

## 6. Conclusions

In this study, a novel framework termed DVIT-UNet was proposed, which integrates a ViT with a dilated convolution block for tea plantation recognition from high-resolution UAV images, thus improving feature extraction. DVIT-UNet significantly outperformed other popular models across multiple accuracy metrics, therefore confirming its effectiveness. The main conclusions of this research, as a result, can be summarized as follows:

The improvement in IoU was particularly pronounced, clearly showing that DVIT-UNet provides superior boundary-delineating through its dual-module design. The ViT component captures global semantic dependencies within images and, in doing so, overcomes the restricted receptive field that limits conventional CNNs. At the same time, the dilated convolution module gathers multi-scale contextual information without compromising spatial resolution. This combination strengthens feature extraction from small-scale tea plots and complex boundaries. As a result, DVIT-UNet achieves better discrimination of fine-grained landcover classes and performs especially well in defining tea plantation boundaries and heterogeneous intra-plantation patterns.

For the precise monitoring, dynamic management, and automated assessment of the world’s tea plantation resources, the suggested DVIT-UNet framework is both technically sound and scientifically significant. Multi-temporal data may be used in future studies to lessen recognition difficulties in areas with bare patches, cloud shadows, and other local complexities.
